# Teaching evidence-based medicine in medical education: data on barriers and requirements in non-university and university hospitals

**DOI:** 10.1186/s12909-025-07887-0

**Published:** 2025-10-02

**Authors:** Larissa Luchsinger, Anne Berthold, Monika Brodmann Maeder, Harald Mayer, Christoph Steinacker, Daniel von Langen, Michael Siegrist

**Affiliations:** 1 ETH Zurich, Institute for Environmental Decisions (IED), Consumer Behavior Group, Universitätstrasse 22, Zurich, CH-8092 Switzerland; 2 Swiss Institute for Medical Education SIWF, Bern, Switzerland; 3 Austrian Medical Chamber, Vienna, Austria

**Keywords:** Evidence-based medicine, Postgraduate medical training, Teaching, Questionnaire

## Abstract

**Background:**

Evidence-based medicine (EBM) is an essential component of medical practice, combining the best available scientific evidence with clinical expertise to support high-quality patient care. There is limited information on how EBM is integrated into postgraduate medical education programs. This study investigates differences in EBM training between university and non-university hospitals and across specialties in Switzerland and Austria.

**Methods:**

Data were collected through annual nationwide surveys evaluating the quality in postgraduate medical education in Switzerland and Austria. A total of 13,659 residents (Switzerland: 9,683; Austria: 3,976) responded to a paper-based questionnaire, resulting in response rates of 71% for Switzerland and 44% for Austria. The questionnaire contains a five-item EBM-scale measuring different aspects of EBM training on a 6-point Likert scale. Residents also answered questions related to scientific publishing. Comparisons were made between university hospitals and other institutions within and between both countries, as well as across specialties within hospital types and countries. Descriptive statistics were reported, and differences were analyzed using the Mann-Whitney U and Chi-square test.

**Results:**

In both countries, residents at university hospitals gave overall significantly higher ratings on the EBM-scale than those at non-university hospitals, with more pronounced differences observed in Austria. Moreover, residents in university hospitals reported higher engagement in scientific activities. Specialty comparisons revealed that internal medicine received the highest EBM ratings among university hospitals. In both countries, there were no significant differences between the two specialities anaesthesia and pediatrics across university and non-university hospitals.

**Conclusions:**

Data from residents of two countries indicate potential to improve EBM training in postgraduate medical education in non-university hospitals, and reveal differences across specialities, suggesting the need for further research to identify the factors underlying these differences across hospital types and clinical contexts.

## Introduction

Evidence-based medicine (EBM) is an empirical approach to medical practice that integrates the best available scientific evidence with clinical expertise from systematic research [1]. It is further defined as the “conscientious, explicit, and judicious use of current best evidence in making decisions about the care of individual patients” [1]. EBM seeks to connect the medical literature with patient care while taking into account the patient’s preferences into the decision-making [2]. Five key steps guide the process of EBM [2, 3]: 1) Define a clearly formulated question based on a patient’s problem, 2) search and gather information from the literature, 3) critically assess the literature for its practicality and validity, 4) apply the findings in clinical practice, and 5) evaluate the effectiveness of the process.

Understanding and practicing EBM is essential for healthcare professionals to provide a high standard in medical care to find the best treatment available for a patient or a disease [4]. According to the literature, teaching actitivies and training courses regarding EBM are implemented in several countries in different specialities in undergraduate and postgraduate programs [5–8]. One important format in EBM training are Journal Clubs [9, 10]. Journal Clubs help to increase reading comprehension and the ability to use findings, and, further, enhance critical appraisal and facilitate the exchange of ideas. The literature suggests that enhancing residents’ EBM skills requires the integration of EBM into both didactic curriculum [11] and clinical practice [10, 12, 13]. In general, residents express a high interest in receiving EBM training [14] and a high perception of the relevance of EBM [15]. Further, a study found that high self-rated EBM capability beliefs were strongly correlated with more frequent EBM use [16]. However, there are several barriers to its implementation in everyday clinical practice. The main barriers identified in the literature are lack of time [17], lack of priority or interest [17–19], limited access to medical information [18], and lack of knowledge and skills, such as low expertise in searching for electronic information resources and difficulties in how and when to stop searching or formulating a clear clinical question [17, 18, 20, 21]. These barriers do not only apply to the residents but also to their trainers. Moreover, team dynamics, learning culture and the work environment are mentioned as barriers as well [18, 20].

Although EBM is widely discussed in the literature, most studies mainly focus on the barriers and attitudes of doctors towards EBM without examing how EBM is applied in postgraduate medical education programs. This article aims to investigate how EBM is assessed in different types of hospitals and across specialities in two countries. The results further help to identify patterns and potential areas for improvement.

## Methods

### Setting

In Switzerland and Austria, the postgraduate training programs for residents last between five to six years on average. In order to obtain their specialist title, residents can attend postgraduate training programs in an university hospital or in another hospital or institution (e.g., outpatient clinic or family doctor’s practice). In both countries, residents have the opportunity to change hospitals or rotate within the medical institutions to different positions in the same or in other specialties. In Switzerland, there are five official university hospitals [22], while Austria has three public university hospitals (Vienna, Graz, Innsbruck).

### Study sample

The present data were collected in 2023 as part of the annual quality assessment of training sites delivering postgraduate medical education in Switzerland [23] and Austria [24]. This annual survey has been conducted in Switzerland since 2003 [23, 25], while in Austria it was conducted for the first time in 2023.

In both countries, the residents’ evaluation of their training site is assessed with the same paper-pencil questionnaire. They receive the questionnaires directly from the directors of the training sites. The questionnaire is anonymous and can only be linked to the training site, not to a specific person. The residents return the questionnaires directly via mail to ETH Zurich.

In Switzerland, 9683 residents returned the questionnaire, which represents a response rate of 71%. In Austria, 3976 residents filled out the questionnaire, which resulted in a response rate of 44%. One reason for this relatively lower rate might be that the questionnaire was yet not as established as in Switzerland as the survey was conducted in Austria for the first time in 2023. Studies show that mail survey response rates are higher in general compared to online surveys [26], and response rates tend to be lower for physicians compared to non-physicians [27]. In contrast, other national surveys with surgical residents have reported an average response rate of 43% [28].

### Measurements

The questionnaire for the annual quality assessment of training sites consists of various questions divided into eight scales related to the training site and working conditions [23]. One scale of these scales is the *evidence-based medicine* scale (EBM-scale). All eight scales, including the EBM-scale, were developed in 2003 in collaboration with Swiss directors of medical institutions and residents, and have been revised several times over the years [23]. For the survey conducted in Austria, the same questionnaire was used with minor adjustments and was approved by the Austrian Medical Chamber. The EBM-scale consists of the following five items: (1) *I am taught to evaluate scientific publications*, (2) I *am taught to apply the findings of a scientific study during the specific treatment of a patient*, (3) *At our institution*,* therapies and diagnostic procedures are regularly discussed based on the current scientific literature*, (4) *At our institution*,* we implement relevant findings from the current scientific literature in everyday clinical practice*, and, (5) *I have access to the most important scientific journals (online or print)*. For answering the questions, a 6-point Likert Scale is used ranging from 1 = does not apply at all to 6 = fully applies. The scale is built by calculating the mean of all five items. Cronbach’s Alpha of the scale was good (Switzerland: α = 0.89, Austria: α = 0.86). In addition, the questionnaire contains two questions in relation to scientific publishing: 1) *Do you aim to publish scientifically?*, and 2*) Do you have the opportunity to work on a scientific publication?.* Regarding both questions, residents could answer with ‘yes’ or ‘no’. These two questions have been part of the questionnaire since 2003 and were developed in workshops to gather additional information on scientific publishing.

### Data analysis

All analyses were conducted in SPSS 28.0. The results include the following comparisons: Differences (a) between university hospitals and other institutions within each country, (b) between Switzerland and Austria within each type of hospital, and, (c) across specialties within each hospital type and country. First, we used the Kolmogorov-Smirnov test to assess if our data was normally distributed. As the EBM-scale scores and its five individual items were not normally distributed, non-parametric tests were applied. We performed the Mann-Whitney U test to analyze the differences regarding the ratings of the EBM-scale and its five items. Descriptive statistics including medians, interquartile ranges, and means were reported. For the nominal variables on scientific publishing, we conducted a Chi-Square Test and calculated Cramers *V* to determine the strength of association. To illustrate the differences across specialities, we calculated the means and confidence intervals.

## Results

Table 1 illustrates that in both countries, Switzerland and Austria, the ratings of residents from university hospitals regarding the EBM-scale and its five items were significantly higher compared to those of residents from other institutions (Switzerland: all *U* < 8512018.5, all *Z* < −7.02, all *p* <.001; Austria: all *U* < 837566.5, all *Z* < −7.36, all *p* <.001). In Switzerland, the largest difference among the five EBM items was found for EBM5 (*U* = 7940570.5, *Z* = −11.66, *p* <.001), whereas in Austria, the largest difference was observed for EBM1 (*U* = 656317, *Z* = −14.59, *p* <.001). According to the data, this difference seem to be more pronounced in Austria. In addition, significant differences also emerged between the two countries across both types of hospitals. The largest difference was found between non-university institutions, with Swiss institutions scoring significantly higher on all five EBM items and the EBM-scale (all *U* < 9831618.5, all *Z* < −12.59, all *p* <.001). Furthermore, the differences between university hospitals in Switzerland and Austria were less pronounced but also significant across all aspects (all *U* < 778624, all *Z* < −2.29, all *p* <.05). Notably, the majority of residents are working at other institutions (CH:72%, A:82%) compared to university hospitals.

**Table 1:** Descriptive statistics of university hospitals and other institutions for the EBM-scale and its five items: median, interquartilrange (IQR) and mean

	Switzerland				Austria			
	university hospitals (*n* = 2714)		all other institutions (*n* = 6969)		university hospitals (*n* = 615)		all other institutions (*n* = 3361)	
	*Median (IQR)*	*Mean*	*Median (IQR)*	*Mean*	*Median (IQR)*	*Mean*	*Median (IQR)*	*Mean*
I am taught to evaluate scientific publications. (EBM1)	4.0 (3.0–5.0)	4.2	4.0 (3.0–5.0)	3.9	4.0 (2.0–5.0)	3.7	2.0 (1.0–4.0)	2.6
I am taught to apply the findings of a scientific study during the specific treatment of a patient. (EBM2)	4.0 (3.0–5.0)	4.2	4.0 (3.0–5.0)	4.0	4.0 (2.0–5.0)	3.8	3.0 (2.0–4.0)	2.9
At our institution, therapies and diagnostic procedures are regularly discussed based on the current scientific literature. (EBM3)	5.0 (4.0–6.0)	4.8	5.0 (4.0–6.0)	4.6	5.0 (4.0–6.0)	4.5	4.0 (3.0–5.0)	4.0
At our institution, we implement relevant findings from the current scientific literature in everyday clinical practice. (EBM4)	5.0 (4.0–6.0)	4.8	5.0 (4.0–6.0)	4.6	5.0 (4.0–6.0)	4.7	4.0 (3.0–5.0)	4.2
I have access to the most important scientific journals (online or print). (EBM5)	6.0 (5.0–6.0)	5.3	5.0 (4.0–6.0)	5.0	6.0 (4.0–6.0)	4.9	4.0 (3.0–6.0)	4.0
EBM-scale	4.8 (4.0–5.6)	4.7	4.6 (3.6–5.2)	4.4	4.6 (3.4–5.4)	4.3	3.6 (2.6–4.6)	3.6

Regarding the overall ratings of the five items of the EBM-scale, items EBM3 to EBM5 received higher scores, while EBM1 and EBM2 were rated lowest. This findings suggests that while residents have access to scientific literature, they may not feel confident in their ability to evaluate or apply it, particulary in non-university hospitals. Also, the pattern of results is of special importance since the first two items (displaying the biggest differences) are strongly related to the classical understanding of EBM.

Table 2 shows the residents’ responses to the two questions related to scientific publishing, differentiated by university hospitals and other institutions. The results show a significant gap between university hospitals and other institutions regarding their share of residents with publication goals and opportunities to work on a publication. The difference is especially notable in Austria. In general, residents from university hospitals more likely aim to publish scientifically compared to those from other institutions (Switzerland: *χ*^*2*^(1) = 167.943, *p* <.001, Cramers *V* = 0.13; Austria: *χ*^*2*^(1) = 535.083, *p* <.001, Cramers *V* = 0.37). Further, the share of residents who have the opportunity to work on publications is cleary higher in university hospitals than in other institutions (Switzerland: *χ*^*2*^(1) = 445.754, *p* <.001, Cramers *V* = 0.22; Austria: *χ*^*2*^(1) = 417.651, *p* <.001, Cramers *V* = 0.33). This is the case in both countries; however, Cramers *V* shows descriptively a stronger association between the type of hospital and the two questions in Austria. In addition, we examined if there were differences between the two countries across the two types of hospitals. The results indicate that the Swiss residents are significanly more likely to publish scientifically compared to residents from Austria (university hospitals: *χ*^*2*^(1) = 23.342, *p* <.001, Cramers *V* = 0.08; other institutions: *χ*^*2*^(1) = 474.442, *p* <.001, Cramers *V* = 0.22). Moreover, Swiss residents reported more frequently than Austrian residents that they have the opportunity to work on publications (university hospitals: *χ*^*2*^(1) = 2.254, *p* <.001, Cramers *V* = 0.03; other institutions: *χ*^*2*^ (1) = 284.188, *p* <.001, Cramers *V* = 0.17). A closer look at the test parameters indicate that these findings further support the pattern observed in Table 1, namely that the differences between Switzerland and Austria are especially evident among non-university institutions, whereas the gap is smaller in the university hospital setting.

**Table 2:** Residents’ responses to the scientific publishing questions differentiated by university hospitals and other institutions

	Switzerland				Austria			
	university hospitals (*n* = 2714)		all other institutions (*n* = 6969)		university hospitals (*n* = 615)		all other institutions (*n* = 3361)	
	Yes (%)	No (%)	Yes (%)	No (%)	Yes (%)	No (%)	Yes (%)	No (%)
Do you aim to publish scientifically?	62	38	48	52	73	27	25	75
Do you have the opportunity to work on a scientific publication?	76	24	52	48	79	21	35	65

**Fig. 1:**
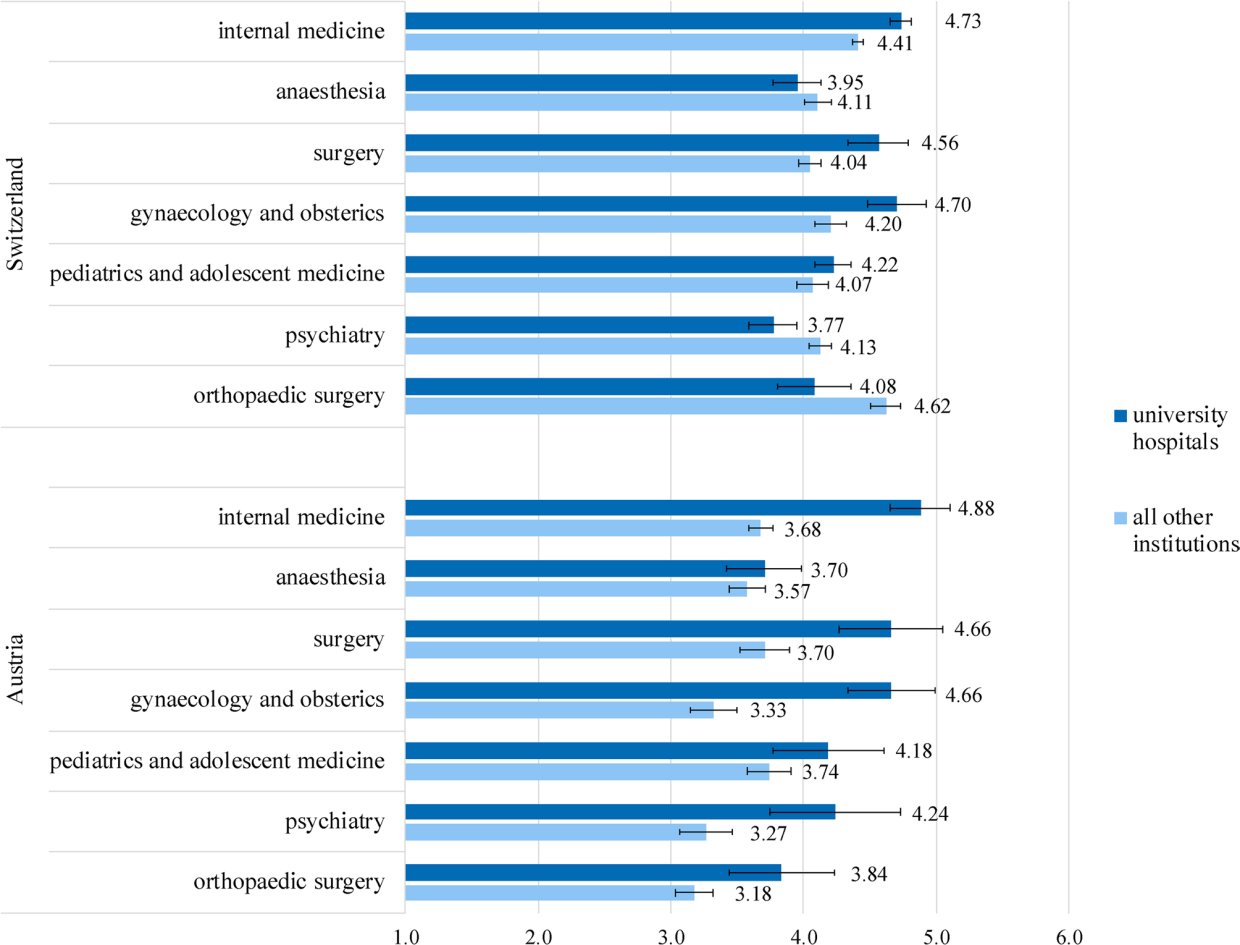
Differences across the seven largest specialities by type of hospital regarding the EBM-scale Notes: The mean values are presented. *N*_CH_=418–2769, *N*_A_=168–849. Error bars represent the 95% confidence interval

A comparison of the seven largest specialties by type of hospital in Switzerland and Austria revealed differences in the scores of the overall EBM-scale within and between the two countries. Once again, the differences between university hospitals and other institutions were more pronounced in Austria than in Switzerland across five specialties. Two specialities, anaesthesia and pediatrics, do not differ significantly in both countries. In both countries, internal medicine garners the highest EBM evaluations among university hospitals. In Switzerland, orthopaedic surgery stands out as the highest rated specialty in EBM among other institutions, while in Austria, it received the lowest rating. There, pediatrics was evaluated highest among the other institutions (Fig. 1).

## Discussion

This article presents and discusses the findings of the assessment of the Evidence based medicine (EBM) scale, which is evaluated in the nationwide annual assessment of postgraduate medical education in Switzerland and Austria. The aim was to better understand how well EBM is integrated into postgraduate medical education in both countries and across various clinical contexts.

We examined the differences in the assessment of the EBM-scale and its five items between university hospitals and other institutions. In both countries, university hospitals received significantly higher ratings than non-university hospitals. Overall, the findings indicate that although residents generally have access to scientific articles, they feel less qualified to evaluate them. Notably, two of the five items of the EBM-scale, EBM1 (I am taught to evaluate scientific publications) and EBM2 (I am taught to apply the findings of a scientific study during the specific treatment of a patient), which most closely align with with Sackett’s five key step of EBM [2], were rated lowest. Additionally, residents in university hospitals are more likely to engage in scientific activities, such as aiming to publish and having opportunities to work on scientific publications. Further, this research presents the differences in the assessment of the EBM-scale of the seven largest specialties in Switzerland and Austria. Regarding university hospitals, internal medicine garners the highest EBM evaluations in both countries. For non-university hospitals, orthopaedic surgery emerges as the highest rated speciality in EBM in Switzerland, while in Austria, pediatrics ranks highest.

When comparing the two countries, Austria shows larger differences between university hospitals and other institutions in the EBM ratings, potentially due to structural factors. Institutions in Austria are geographically more dispersed and less locally connected to universities compared to those in Switzerland. Moreover, residents in Austria may have a higher patient load, possibly related to differences in the healthcare system. In Switzerland, patients typically consult a family medicine clinic instead of going directly to hospitals which might reduce the number of patients needing treatments in hospitals. A higher patient load leads to less time available, posing one of the major barriers for EBM [13]. Additionally, differing curricula between specialities and between university hospitals and other institutions may also contribute to these discrepancies.

Based on our findings, potential improvements in EBM practices are mainly evident in non-university hospitals, where the majority of residents work. Additionally, differences were observed across specialties. Although the data presented in this manuscript are limited to two countries, the patterns observed may be relevant to other settings with similar clinical contexts. This manuscript did not explore the underlying causes of these patterns. Therefore, further research is warranted to better understand the factors driving variability in EBM practices and education across hospital types and specialities. Finally, an ongoing evaluation and refinement of EBM practices based on resident feedback may offer insights on how EBM can remain current and effective in clinical practice of postgraduate medical education.

### Limitations

Some limitations need to be discussed. First, the items of the EBM-scale are formulated in general terms in order that residents from different specialities can respond to them. Secondly, the questionnaire does not directly address barriers for teaching EBM, thus, any assumptions and inferences should be considered with caution. Third and last, specialties can only be compared to a limited extent between the two countries due to differences in their curricula.

## Conclusions

To conclude, EBM is vital for improving patient outcomes and ensuring high-quality healthcare. To work in an evidence-based manner, residents need to be competent across different domains in EBM, such as knowledge, literature appraisal, and application of evidence. This study reveals gaps in EBM training between university hospitals and non-university institutions in Austria and Switzerland, with university hospitals performing better. These differences are particularly pronounced in Austria, where structural factors may hinder EBM integration in non-university settings. Further research is required to find the underlying causes for these differences and to address these gaps.

## Data Availability

The datasets generated and analysed are not publicly available, as both the residents in Switzerland and Austria are promised every year that the data will not be disclosed.
